# The impact of nanofertilizer on agro-morphological criteria, yield, and genomic stability of common bean (*Phaseolus vulgaris* L.)

**DOI:** 10.1038/s41598-022-21834-9

**Published:** 2022-11-03

**Authors:** Dina M. Salama, M. E. Abd El-Aziz, Essam A. Shaaban, Samira A. Osman, Mohamed S. Abd El-Wahed

**Affiliations:** 1 Vegetable Research Department, National Research Centre, 33 El Bohouth St., Dokki, P.O. 12622, Giza, Egypt; 2 Polymers and Pigments Department, National Research Centre, 33 El Bohouth St., Dokki, P.O. 12622, Giza, Egypt; 3 Pomology Department, National Research Centre, 33 El Bohouth St., Dokki, P.O. 12622, Giza, Egypt; 4 Genetics and Cytology Department, National Research Centre, 33 El Bohouth St., Dokki, P.O. 12622, Giza, Egypt; 5 Botany Department, National Research Centre, 33 El Bohouth St., Dokki, P.O. 12622, Giza, Egypt

**Keywords:** Biochemistry, Genetics, Plant sciences, Nanoscience and technology

## Abstract

The use of agricultural fertilizers is one of the methods to beat the desired enormous increase in universal food production. The application of nanotechnology in agriculture is regarded as one of the promising approaches to elevate crop production. Whereas mineral nutrients play a crucial role in the growth and yield of the common bean. The experiments were conducted to investigate the application effect of micronutrients as nanoparticles (MN-NPs) on the common ben plants. The trial was performed in the field in El-Menofya, Egypt, through two seasons (2019 & 2020) in a randomized complete block design with three replicates and four combinations of MN-NPs (ZnO, MnO_2_ and MoO_3_) with concentrations 0, 10, 20, 30, 40 mg/L as a foliar application. The data exhibited that the foliar application of MN-NPs significantly upgraded the vegetative growth characters, flower number/plant, photosynthetic pigments, and yield. The concentration of 40 mg/L of MN-NPs leads to improving the vegetative growth, flowering number, and yield characteristics of the common bean. While the biochemical components varied in their response to MN-NPs combinations. The recommended MN-NPs concentration to ameliorate the common bean growth and yield was 40 mg/L.

## Introduction

It is anticipated in 2050 that the population of the world will rise to 9.6 billion^1^, which makes improving the yield of crops and grains will be required. Which pushed FAO^2^ to state that the yield of grain must be raised by 70 percent in 2050 to overcome these requests. Whereas the water sources and arable lands are restricted, agricultural fertilizers are the available approach to increase universal food production. So, it had been tended to raise the amount of added fertilizer to progress the yield of crops, but the increase in using traditional fertilizers for a prolonged period has produced large international environmental issues^3,4^. For that, researchers are girding to evolve fertilizers and their efficiency to increase agriculture production, as well as reducing environmental pollution. These nutrients are supplied as fertilizers, which are used to promote the growth and yield of plants. They are split into two main categories; macronutrients include nitrogen, phosphorus, and potassium (NPK), and micronutrients include calcium (Ca), mangnesium (Mn), zinc (Zn), iron (Fe), copper (Cu), molybdenum (Mo). These nutrients are regularly applied either by addition to the soil or as a foliar application^5,6^.

Zn is an essential micronutrient for plants, which used in small amounts for improve the activities of several enzymes and protein^1–3^. its deficiency causes the plants to suffer physiological stress due to its effect on several enzyme systems and metabolic actions Zn^7^. It plays an important role in the metabolism of proteins and carbohydrates, controlling the growth hormone^8^, and the production of tryptophan and growth hormone auxin^9^. Mahajan, Dhoke, and Khanna^10^ found that the growth of *Vigna radiate* and *Cicer arietinum* was improved by using 20 mg/L and 1 mg/L of ZnO-NPs, respectively. Prasad et al.^11^ demonstrated that the germination, growth and pod yield of peanuts was improved by using ZnO-NPs as a foliar than chelated ZnSO_4_.

Whereas manganese plays an essential role in the metabolism of nitrogen as well as photosynthesis where it acts as an electron store and travels them to the chlorophyll centers^12^. Also, it acts as is antioxidant e.g. dismutase to prevent spoilage of plant tissue by free radicals^13^. It is usually used in the range of 1–2 kg/ha^14^. Pradhan et al. showed that 0.05 mg/L of manganese nanoparticles increased the percentage of root and shoot length by 52% and 38% for mung bean plants in comparison to control^15^.

Molybdenum is an essential element for plant growth and biological fixation of nitrogen (BNF) by combining it with enzymes. Mo is important for nitrogen metabolism where it is accountable for the production of ammonium (NH_3_) from nitrogen. Its deficiency causes a lowering in the activity of molybdoenzymes and so decreasing plant growth and yield^16^. Taran et al.^17^ found that soaking chickpea seeds for 1–2 h in Mo-NPs solution ameliorates the growth and yield of chickpea plants.

Nanotechnology is a new trend that is being used in various industrial applications like pharmaceutical, packaging materials, sensors, conductors and agriculture^18,19^. It is participated in many stages of food preparation, from the beginning of cultivation to the packaging of the product for the consumer. Currently, it is used in the agricultural process to achieve the concept of smart agriculture or sustainable agriculture^20^. Where it can be used in the preparation of fertilizers, pesticides, and water purification that are used in agriculture processes as well as in remote sensing^21^. One way to improve crop production and conquer global food requirements is the implementation of nanotechnology in agricultural manure preparation^22–24^. Indeed, nanotechnology has the potency to improve the entire current agrarian and food industry, by improving treatment methods for plant disease, and nutrient use efficiency^26^. Nowadays, nanofertilizers have great attention to be utilized instead of traditional fertilizers, which reached plant tissues via the root or leaves^27^. Where they were more effective in the planting process and lowing environmental impact by decreasing the losses to the surrounding environment^28,29^. Indeed, the NPs shape, size, or dosage affects the plants. Furthermore, the use of nanofertilizer could promote the existing functions or add new ones^30,31^.

DNA imprint is a remarkable molecular marker assay to illustrate the mutagenicity that occurs outstanding in the interplay between chemicals and plants^32^. Different molecular markers for example RAPD, SRAP, and ISSR can be performed to illustrate the variations that occur in DNA^33^. ISSRs have many advantages to being widely used such as; being easier and faster to use, inexpensive, as well no requirement for information about genomic sequences^34,35^. ISSRs have many advantages to being widely used such as; being easier and faster to use, inexpensive, as well no requirement for data about genomic sequences^34^. They are represented by the recognition of the changes that occur in the DNA profiles that may be formed as a new apparition or missing of some bands, or alteration in the intensities of other bands^36^.

The common beans (*Phaseolus vulgaris* L.) are one of the imperative food legumes for humans, which have huge differences in seed characteristics such as shape, volume, and color, besides growth habits, and ripeness. Their seeds are considered an important source of carbohydrates, vitamins, protein, and minerals, especially for developing countries humans besides they are the economic part. Therefore, it is counted in Egypt as one of the best essential vegetable crops planted for the local and export mart^37,38^.

Therefore, this study was conducted to investigate the influence of the combination of micronutrients (Zn, Mn and Mo) in nano-form as a foliar application on the quantity and quality of the common bean plus the genomic DNA stability.

## Materials and methods

### Materials

Common bean seeds (cv. Nebraska) purchased from the Agricultural Research Centre, Egypt. Agriculture traditional fertilizers such as sulfur, calcium superphosphate, potassium sulphate, and ammonium sulfate, bought from Abu Qir Company, Egypt. Manganese nitrate and citric acid were obtained from Sigma-Aldrich Company. Sodium hydroxide, ammonium hydroxide, zinc acetate, and ammonium molybdate were purchased from s.d. Fine-Chem.

### Preparation of nanofertilizer

Zinc oxide nanoparticles (ZnO-NPs) were prepared by refluxing 3.942 g zinc acetate in 1L ethanol containing 1.44 g NaOH at 70 °C for 2 h. The fine white powder was obtained and purified by adding deionized water and then centrifugation for 10 min at 5000 rpm, after that the fine white powder which burned for 2 h at 500 °C to obtain ZnO-NPs^39^.

Manganese oxide nanoparticles (MnO_2_-NPs) were prepared thermally by dissolving Mn(NO_3_)_2_·4H_2_O in water (100 g/50 ml) at 100 °C under vigorous stirring for 10 min, after that the previous solution was heated in an oven to 100 °C for 24 h till black viscous liquid was achieved. The deionized water was added to the previous liquid and centrifugation for 15 min at 7000 rpm. The black particles were washed with deionized water and dried at 100 °C^40^.

Molybdenum oxide nanoparticles (MoO_3_-NPs) were prepared via a sol–gel method, where 11.6 g ammonium molybdate and 3.8 g citric acid were dissolved in distilled water under stirring. The pH was adjusted at 7 by ammonium hydroxide, and then heated to 250 °C for 1 h to get a powder, then burned for 120 min at 500 °C to get MoO_3_-NPs^41^.

### Characterization

A transmission electron microscope (TEM; JEM-1230, Japan) was employed to illustrate the morphology and particle size of the nanoparticles, which operated at 120 kV, magnification of 6 × 10^5^, and a resolution is 0.2 nm. While the geometry and purity of prepared nanoparticles were determined using a Philips X-ray diffractometer (PW 1930 generator, PW 1820 goniometer, and radiation source CuK).

### Experiment layout

Experimental research and field studies on common bean plants obey the guidelines of the Ethics Committee in the National Research Centre.

The ground had been scrubbed, ploughed, and leveled in February, after that, it was divided into plots. The organic manure (cattle manure), calcium superphosphate (15.5% P_2_O_5_), agricultural sulfur, potassium sulphate (48% K_2_O), and ammonium sulphate (20.6% N) were added in the range 47.6 m^3^/ha, 476, 236, 120, and 118 kg/ha, respectively, during the soil preparation. After seeds germination, ammonium sulphate (236 kg/ha) was added. Before the second irrigation, ammonium sulphate and potassium sulphate were applied in quantities 238 and 120 kg/ha, respectively.

The common bean seeds (120 kg/ha) were cultivated on 3 March throughout two seasons 2019 and 2020 in clay ground in El-Monifia governorate, Egypt. Table S1 showed the physical and chemical characteristics of the soil. The samples of soil were collected from various sites of each plot from the depth of 0 to 20 cm, which were analyzed according to Cottenie et al*.*^42^. Two seeds per hole were sowed on one side of the ridge and 30 cm between holes while the distance between the ridges was 60 cm. The harvest was done after three months. The average weekly temperature ranged from 20 to 31 °C during the period of the cultivation (3 Mar–3Jun) as illustrated in Fig. S2.

### The transactions

The common bean plants were sprayed after 20 days of sowing by a combined three nanofertilizer with various concentrations (0, 10, 20, 30, and 40 mg/L) as shown in Table 1. The treatments were performed in the morning (8 am) with one liter of suspended nanomaterials in water (pH 7.2) using a hand-pump garden sprayer. The experiment was set up in a complete randomized block design with three replicates.

**Table 1:** The treatment recipe.

Treatment	ZnO-NPs (mg/L)	MnO_2_-NPs (mg/L)	MoO_3_-NPs (mg/L)
T1 (control)	0	0	0
T2	10	10	10
T3	20	20	20
T4	30	30	30
T5	40	40	40

### Agro-morphological criteria

Three random plants in vegetative growth stage were taken from each dealing (after 45 days of sowing) to determine various vegetative growth factors, for example, the length of plant and root, the numeral of leaves and flowers per plant, besides the weight of fresh and dry for plants. After 90 days of sowing (Harvest stage) three samples were taken to measure the weight and number of seeds and pods per plant, plus shelling percentage, seed index (weight of 100 seeds), the weight of seeds (t/ha) and shoot residues (kg/ha).

### Chemical studies

#### Photosynthetic pigments

The fresh leaves were collected after 45 days of sowing to extract photosynthetic pigments using 85% acetone. The concentrations of chlorophyll a (Chl. a), chlorophyll b (Chl. b), and carotenoid (Cart.) were measured by UV/VIS spectrophotometer (TG 80, Germany) at 663, 644, and 452 nm, respectively, against a blank (85% acetone)^43^.

The concentration of the pigments was measured as µg/ml and calculated using the equations proposed by Jiang et al.^44^.

$${\text{Chl a }} = {1}0.{\text{3 E663 }} - \, 0.{\text{918E644}} \left( {\mu {\text{g}}/{\text{ml}}} \right)$$

$${\text{Chl b }} = {19}.{\text{7E644 }} - { 3}.{87}0{\text{E663}} \left( {\upmu {\text{g}}/{\text{ml}}} \right)$$

$${\text{Caro }} = {4}.{\text{2 E452 }} - \, \left( {0.0{\text{264 Chl a }} + \, 0.{\text{426 Chl b}}} \right) \left( {\upmu {\text{g}}/{\text{ml}}} \right).$$

Then concentrations of chlorophyll and carotenoids were expressed as mg/g fresh weight of plant material.

#### Proximate analysis

Fresh samples of leaves and seeds were dried at 60 °C in the oven till constant weight and then ground for proximate analysis by the standard methods according to AOAC^45^. The moisture content was calculated by weighing the sample before and after drying till a constant weight at 105 °C. The ash was obtained by burning at 550 °C for 6 h in a muffle furnace. The crude protein was measured by the Kjeldahl method (N × 6.25). The fiber content was calculated after digestion in acid (1.25%) then in alkali (1.25%) then extracting by ether. The total energy was calculated by the Atwater factor method [(9 × fat) + (4 × carbohydrate) + (4 × protein)] as described by Nwabueze^46^.

#### Minerals determination

Plant samples were ground and digested with H_2_SO_4_-H_2_O_2_. In the digested solutions, the concentration of potassium and phosphorus were determined by a spectrophotometer, while atomic absorption was used to measure the content of Zn, Mn, Fe and Cu^47^.

### Statistical analysis

The statistical analysis of the obtained data was done according to the procedures described by Snedecor et al.^48^ to determine significant differences using LSD at a 5% level.

### DNA purification and SCoT-PCR analysis

The plant genomic DNA was extracted and purified from leaves of the common bean using Gene Jet Plant Genomic DNA purification Mini Kits (Thermo scientific K0791). NanoDrop 1000 spectrophotometer (Thermo Scientific) was used to measure the quantitation of pure genomic DNA. Eight SCoT Primers with GC content between 50 and 72% (Table 2) were used in PCR reaction^49^. PCR amplification was achieved using Thermocycler (Bio-Rad). The PCR was programmed as follows: an initial denaturation one cycle for 3 min at 94 °C, followed by 35 cycles for 1 min at 94 °C, then for 1 min at 50 °C, and 2 min at 72 °C; the final extension at 72 °C for 5 min (one cycle) then held at 4 °C. Agarose gel was placed in 1X TAE buffer (1.2%) for running the amplified products at 100 V for 2 h. The Gel Documentation (Bio-Rad) was used for photographing the gel and then analyzed by the Total Lab program to find out the molecular size of each band.

**Table 2:** Sequences of SCoT primers.

Ser. No	Primer	Sequence (5–3)	GC%
1	SCoT-2	CAACAATGGCTACCACCC	56
2	SCoT-4	CAACAATGGCTACCACCT	50
3	SCoT-5	CAACAATGGCTACCACGA	50
4	SCoT-22	AACCATGGCTACCACCAC	56
5	SCoT-26	ACCATGGCTACCACCGTC	61
6	SCoT-34	ACCATGGCTACCACCGCA	61
7	SCoT-36	GCAACAATGGCTACCACC	56
8	SCoT-30	CCATGGCTACCACCGGCG	72

The Genomic Template Stability (GTS, %) was calculated as the following:

$$\hbox{GTS}=100-\left(\frac{100a}{n}\right)$$

where, (a) is the average number of the differences in DNA profiles, and (n) is the number of bands that were selected through control profiles of DNA.

### Proteins banding pattern

Sodium Dodecyl Sulfate Polyacrylamide Gel Electrophoresis technique (SDS-PAGE) was performed according to Tsugama et al.^50^ to separate the proteins based on their molecular weight. The water-soluble proteins (W.S.P.) were extracted from the leaves of common bean plants in the seedling and flowering growth stage. BLUltra Prestained Protein Ladder (GeneDirex, Cat No. PM001-0500) was used as a protein marker. The extracted proteins were electrophoresed by vertical electrophoresis (Cleaver, UK), on 12% protein separating gel.

### Toxicity test

In an oven at 105 °C, the common bean seeds were well dehydrated and then milled to a fine powder. An extract was obtained from this powder using water at two concentrations 0.1% and 1.0%. The toxicity of the samples was performed by using Microtox analyzer 500 (USA)^51^.

## Results

### Characteristics of nanofertilizer

**Figure 1:**
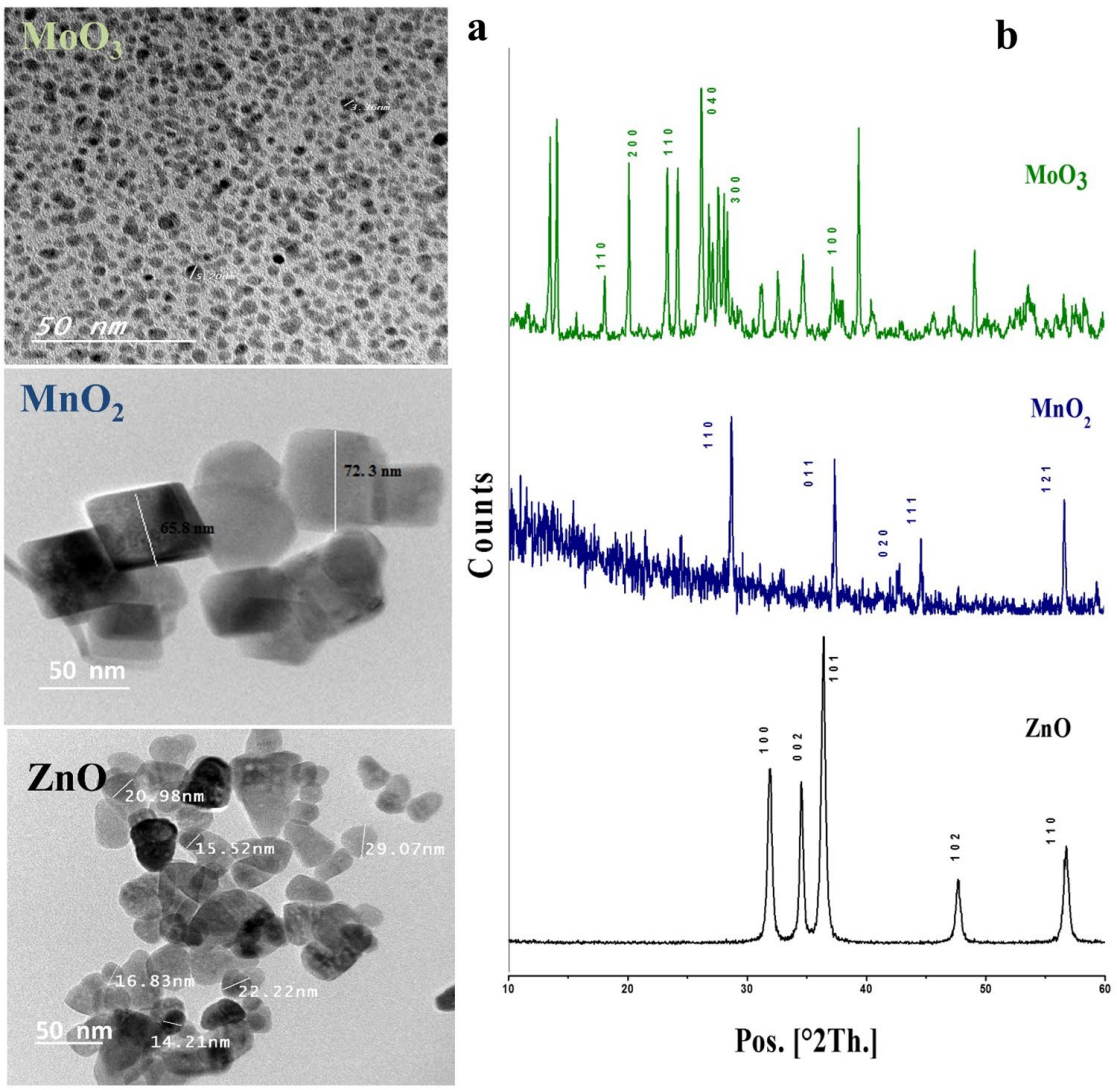
TEM image (**a**) and XRD pattern (**b**) of ZnO, MnO_2_, and MoO_3_-NPs.

Figure 1a illustrated the morphology of the prepared nanoparticles, which demonstrated that they had particle sizes less than 100 nm. TEM image of ZnO-NPs showed spherical particles with an average particle size of less than 50 nm, while MnO_2_-NPs had a uniform diamond shape with an average particle size of 70 nm. Instead, the MoO_3_-NPs showed the smallest size with a mean particle size of 10 nm.

Figure 1b showed the crystal structure of the ZnO, MnO_2_, and MoO_3_–NPs via using X-ray diffraction. The main peaks of ZnO-NPs obtained at 2θ = 32, 34.4, 36.4, 47.7°, and 56.7° are related to plans (100), (002), (101), (102) and (110), respectively^39^. Moreover, the peaks at 2θ (28.7 and 37.3°), (24.8 and 65°) and (42.82, 46.02, 56.65, and 59.3°) are corresponding to α-MnO_2_, γ-MnO_2,_ and β-MnO_2_, respectively^52^. While the pattern of MoO_3_–NPs illustrates the peaks at 2θ = 17.9, 19.9, 26.1, 34.5, 39.3, and 49° resemble the orthorhombic crystal structure of MoO_3_^53^. The XRD patterns emphasize the pureness of the prepared oxide nanoparticles.

### Vegetative growth characters

The foliar implementation of MN-NPs as a fertilizer has significantly affected the vegetative outgrowth traits of the common bean as expressed as plant and root length, number of leaves and flowers per plant, and fresh and dry weight for plant throughout both seasons 2019 & 2020 in comparison with the control (Table 3). The improvement of vegetative characters (length of plant and root, number of leaves per plant, plant weight for plants (fresh and dry weight)), and the numeral of flowers/plants were associated with raising the concentricity of MN-NPs. The concentration of 40 mg/L gave the greatest values of the vegetative characters where the plant length was (57.3–59.0 cm), root length was (28.0–27.0 cm), the number of leaves was (35.3–34.3) fresh weight was (156.4–159.6 g), dry weight was (29.8–30.3 g), and the numeral of flowers/plants was (84.0–85.0) during both seasons 2019 & 2020, respectively. The obtained consequences recorded a significant effect in the vegetative characters at LSD 5% which are as follows: plant length (1.58 and 1.89), root length (2.09 and 1.42), number of leaves per plant (2.41 and 2.12), number of flowers per plant (2.78 and 2.84), fresh weight for plants (7.87 and 8.34), and dry weight for plants (1.53 and 1.59), during agriculture seasons 2019 and 2020.

**Table 3:** Effect of MN-NPS on vegetative growth characters of common bean plants during two successive seasons 2019 and 2020.

Treatment	Plant length (cm)		Root length (cm)		No. leaves/plant		No. flowers/plant		Fresh plant weight (g)		Dry plant weight (g)	
	2019	2020	2019	2020	2019	2020	2019	2020	2019	2020	2019	2020
T1	53.3	53.7	16.3	16.8	29.3	29.7	59.3	63.3	130.9	131.3	22.9	24.6
T2	47.7	48.3	17.8	17.2	29.7	30.3	62.7	64.3	133.3	131.4	24.6	24.1
T3	53.7	54.7	22.3	21.3	29.3	30.3	64.3	66.7	143.0	145.0	26.2	26.1
T4	55.5	56.3	24.3	23.3	30.3	31.3	68.7	70.3	150.2	153.0	26.9	27.6
T5	57.3	59.0	28.0	27.0	35.3	34.3	84.0	85.0	156.4	159.3	29.8	30.3
LSD at 5%	1.58	1.89	2.09	1.42	2.41	2.12	2.78	2.84	7.87	8.34	1.53	1.59

### Common bean yield

The data in Table 4 displayed that the implementation of MN-NPs (ZnO, MnO_2_, and MoO_3_) significantly increased the yield and its components of the common bean plant through seasons 2019 and 2020, in comparison to control. Increasing the concentration led to enhancement of the yield and its components except for shelling percentage, this was related to 10 mg/L concentration. The highest MN-NPs concentration (40 mg/L) gave the greatest values of the yield and its components characters, where the number of pods/plant (37.3–38.7), weight of pods/plant (58.5–60.7 g), number of seeds /plant (10.6–105.0), seed yield (2.24–2.34 t/ha) and shoot residue (913.0–931.3 kg/ha) during the two seasons 2019 & 2020, respectively. From these we found that the increasing percentage of the numeral of pods/plant is (89.3–93.5%), the weight of pods/plant is (26.6–29.2), the numeral of seeds per plant is (58.7–51.5), the shelling percentage is (23.2–25.5), seed yield is (55.6–61.4) and shoot residue is (51.4–49.4) through the seasons 2019 & 2020, respectively.

**Table 4:** Effect of MN-NPs on yield and its components of common bean plant during two seasons.

Treatment	No. seeds/ plant		No. pods/ plant		Weight (g)/ plant						Shelling percentage		Seed index (100 seeds weight (g))		Seeds yield (t/ha)		Shoot residues (kg/ha)	
					Poods		Seeds		Shoot residues									
	2019	2020	2019	2020	2019	2020	2019	2020	2019	2020	2019	2020	2019	2020	2019	2020	2019	2020
T1	67.0	69.3	19.7	20.0	46.2	47.0	26.3	26.4	11.0	11.3	56.7	56.0	39.1	37.9	1.44	1.45	603.2	623.3
T2	90.0	94.0	28.0	28.7	53.9	54.5	39.5	38.5	13.1	12.8	73.4	70.7	43.9	41.1	2.17	2.12	720.5	702.2
T3	100.3	101.0	33.0	34.0	55.9	56.8	33.3	33.0	14.4	14.6	59.6	58.6	33.1	32.6	1.83	1.82	792.0	801.2
T4	103.0	102.3	33.7	34.3	56.4	59.0	38.5	42.3	14.5	15.1	68.1	71.7	37.3	41.6	2.12	2.32	799.3	832.3
T5	106.3	105.0	37.3	38.7	58.5	60.7	40.8	42.6	16.6	16.9	69.9	70.3	38.5	40.8	2.24	2.34	913.0	931.3
LSD at 5%	3.64	4.75	1.02	0.80	2.67	3.94	1.73	1.12	0.67	0.53	3.54	3.39	2.27	2.88	0.10	0.06	36.66	29.24

### Chemical studies

#### Photosynthetic pigments

**Figure 2:**
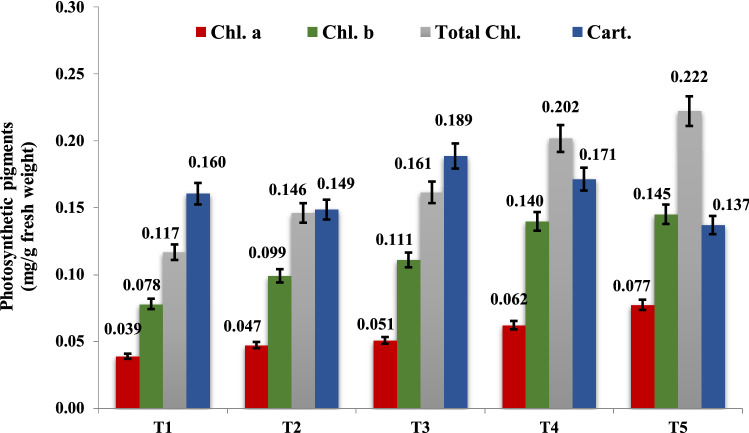
Effect of MN-NPs on the average value of the two seasons for the Chl a, Chl b, total chlorophyll, and carotenoids.

It appeared from Fig. 2 that the implementation of MN-NPs remarkably increased the content chl. a, chl. b, total chl., and carotenoid (photosynthetic pigments) in the common bean plant in compression to control through seasons 2019 and 2020. Increasing photosynthetic pigments content was related to enhancing MN-NPs concentration. Application of 40 mg/L (T5) gave the maximum values of chl. a, chl. b, and total chl. content, while the uppermost content of carotenoids was found with 20 mg/L of MN-NPs (T3).

#### Proximate components

The micronutrient NPs appeared a significant increment in proximate components of common bean leaves such as carbohydrate, protein, lipid, fiber, ash, and total energy in comparison with the control during two seasons (Table 5). The MN-NPs at a concentration of 40 mg/L resulted in a significant increase in the carbohydrate percentage (43.4 and 42.9%) as well as the total energy (285.1 and 284.6 kcal/g) in the common bean leaves compared to other concentrations during two seasons (Table 5). On the contrary, the protein percentage (20.7 and 20.5%) gave the best value at a concentration 20 mg/L of MN-NPs followed by plants treated with a concentration 30 mg/L of MN-NPs. The lipid (4.91 and 5.0%) and ash (18.7 and 19.1%) content in leaves of common bean increased with a concentration 30 mg/L of MN-NPs as compared to other concentrations. The highest percentage of moisture (7.33 and 7.37%) and fiber (11.93 and 11.97%) was recorded with plants grown without MN-NPs.

**Table 5:** Effect of micronutrients nanoparticles on proximate components of common bean**.**

Treatment	Moisture (%)		Carbohydrate (%)		Protein (%)		Lipid (%)		Fiber (%)		Ash (%)		Total energy (Kcal/g)	
	2019	2020	2019	2020	2019	2020	2019	2020	2019	2020	2019	2020	2019	2020
**Leaves**														
T1	7.33	7.37	39.5	39.6	18.2	18.3	4.73	4.80	11.93	11.97	18.4	18.0	273.1	274.8
T2	6.56	6.52	39.1	39.0	19.5	19.3	4.65	4.68	11.45	11.30	18.7	19.1	276.5	275.7
T3	7.07	7.10	38.0	37.5	20.7	20.5	4.82	4.87	11.57	11.38	17.8	18.7	278.2	275.7
T4	7.17	7.12	37.7	37.4	20.3	20.2	4.91	5.00	11.15	11.10	18.7	19.1	276.4	275.6
T5	6.64	6.59	43.4	42.9	18.4	18.6	4.20	4.29	11.06	11.06	16.3	16.6	285.1	284.6
LSD at 5%	0.23	0.25	0.19	0.37	0.24	0.36	0.35	0.36	0.72	0.69	0.98	51.03	3.57	3.41
**Seeds**														
T1	6.4	6.3	39.2	39.4	20.2	20.0	4.00	3.90	13.1	13.2	17.1	17.2	273.6	272.7
T2	9.6	9.5	43.6	43.0	19.2	19.3	5.65	5.60	12.5	12.3	9.4	10.3	302.3	299.3
T3	10.4	10.5	44.5	43.5	17.9	18.0	5.64	5.63	12.3	12.1	9.3	10.4	300.2	296.3
T4	10.6	10.6	45.4	44.9	17.7	17.8	5.69	5.73	11.6	11.6	9.0	9.4	303.5	302.1
T5	11.9	11.6	40.3	40.7	18.0	17.9	5.66	5.68	12.5	12.2	11.6	11.9	284.0	285.5
LSD at 5%	0.18	0.29	1.56	2.00	0.89	0.79	0.06	0.11	1.93	2.02	3.11	2.82	7.22	7.58

In addition, Table 5 shows that the MN-NPs significantly affect LSD at 5% in proximate components of common bean seeds during the 2019 and 2020 seasons. Micronutrient as NPs with concentration 30 mg/L (T4) were more effective on the content of carbohydrate (45.4 and 44.9%), lipid (5.69 and 5.73%) and total energy (303.5 and 302.1 kcal/g), while 40 mg/L (T5) gave the best values of moisture percentages (11.9 and 11.6%), during two successive seasons 2019 and 2020. On the other hand, the increasing seeds content of protein (20.2 and 20.0%), fiber (13.1 and 13.2%), and ash (17.1 and 17.2%) were related to (T1) control during two cultivated seasons 2019 and 2020.

#### Minerals content

Data in Table 6 displayed that the minerals content in the leaves and seeds of common bean plants were significantly enhanced with micronutrient NPs application compared with control during (2019 & 2020) seasons. The content of phosphorus (0.30 and 0.31%) and potassium (3.43 and 3.43%) in leaves of common bean plants appeared the highest value with control as compared to MN-NPs. Contrariwise, the content of leaves of zinc (31 and 30 mg/L),and manganese (63 and 62 mg/L) enhanced with the lowest concentration of MN-NPs (10 mg/L) followed by plants treated with MN-NPs at a concentration of 20 mg/L, during two seasons 2019 and 2020. The concentration of MN-NPs (20 mg/L) improved the content of iron in leaves (760.0 and 756.6 mg/L) and in descending order the plants processed with MN-NPs at a concentration of 10 mg/L, during two agriculture seasons 2019 & 2020. The nitrogen percentage (3.31 and 3.31%) was boosted with the application of MN-NPs at a concentration of 30 mg/L as compared with other treatments.

**Table 6:** Effect of nanofertilizer on minerals of common bean leaves and seeds during two seasons.

Treatment	N %		P %		K %		Zn (mg/L)		Mn (mg/L)		Fe (mg/L)		Cu (mg/L)	
	2019	2020	2019	2020	2019	2020	2019	2020	2019	2020	2019	2020	2019	2020
**Leaves**														
T1	2.90	2.87	0.30	0.31	3.43	3.43	20.00	19.33	44.0	43.5	611.3	609.9	8.73	8.57
T2	2.83	2.79	0.21	0.20	2.90	2.65	31.00	30.00	63.0	62.0	727.3	724.6	13.07	12.97
T3	3.11	3.07	0.29	0.31	2.64	2.87	20.90	20.67	59.7	58.8	760.2	756.6	13.00	12.67
T4	3.31	3.31	0.26	0.29	3.05	3.24	19.70	19.40	56.3	55.6	725.3	721.3	13.00	12.63
T5	3.22	3.18	0.29	0.31	3.02	2.66	18.40	18.20	48.5	49.2	600.1	597.8	11.30	11.47
LSD at 5%	0.02	0.04	0.04	0.05	0.14	0.13	0.53	0.54	1.29	1.48	8.19	9.02	0.38	0.57
**Seeds**														
T1	2.93	2.87	0.32	0.31	2.51	2.45	20.2	20.0	4.1	4.2	44.4	43.7	9.1	9.3
T2	3.22	3.19	0.25	0.24	2.40	2.37	23.5	23.2	5.9	5.8	17.3	18.0	12.0	11.8
T3	3.05	3.02	0.26	0.26	2.44	2.38	23.1	23.2	6.2	6.0	21.8	22.4	10.7	10.8
T4	2.84	2.77	0.26	0.28	2.57	2.48	26.4	26.1	6.0	6.0	20.7	21.5	10.5	10.7
T5	2.81	2.71	0.32	0.31	2.15	2.21	21.5	21.7	4.3	4.5	20.4	20.5	9.1	9.3
LSD at 5%	0.09	0.13	0.01	0.02	0.12	0.13	0.52	0.45	0.10	0.09	0.70	0.67	0.24	0.28

The effect of different concentrations of MN-NPs on endogenous minerals (Nitrogen, phosphorus, potassium, zinc, manganese, iron, and copper) in seeds of the common bean plant was presented in Table 6. Data was obvious that the foliar application of MN-NPs had a significant effect on the minerals of common bean seeds. The best increment of nitrogen (3.22 and 3.19%), and copper (12 and 11.8 mg/L) were obtained in seeds of common bean plants sprayed with MN-NPs at a concentration of 10 mg/L followed by plants supplied with MN-NPs at a concentration of 20 mg/L, during two cultivated seasons 2019 and 2020. Conversely, the highest content of manganese in seeds was recorded with MN-NPs at a concentration of 20 mg/L followed by plants sprayed with MN-NPs at a concentration of 30 mg/L. The content of potassium (2.57 and 2.48%) and zinc (26.4 and 26.1 mg/L) was significantly increased as a result of using MN-NPs as a foliar fertilizer at a concentration of 30 mg/L as compared to other concentrations, during two successive seasons 2019 and 2020. The maximum content of phosphorus (0.32 and 0.31%) was achieved with the highest concentration of MN-NPs (40 mg/L) as compared to other concentrations, during two agriculture seasons 2019 and 2020.

### Effect of different nanofertilizer on genomic DNA using SCoT-PCR Marker

**Figure 3:**
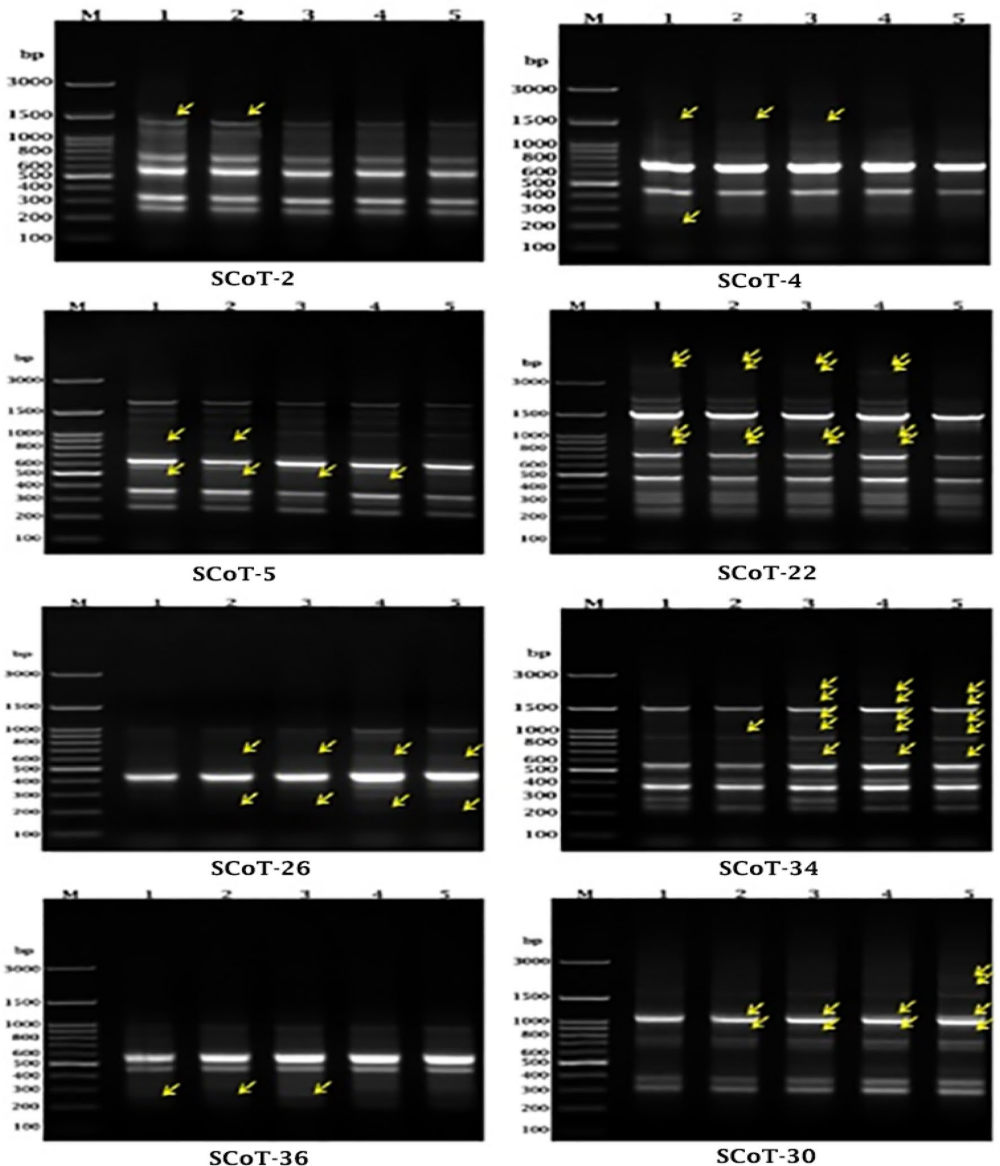
The effect of MN-NPs concentrations on the genomic DNA for common bean by SCoT-PCR. M: DNA ladder 100 bp, 1: T1, 2: T2, 3: T3, 4: T4, 5: T5.

Eight primers were utilized for the SCoT amplification to determine the variation in DNA fingerprints of *Phaseolus Vulgare* L. exposed to various concentrations of MN-NPs and the control (Figs. 3, and Table 7). The original figure obtained from SCoT-PCR was illustrated in S3. The eight SCoT primers distinguished 88 DNA fragments in controls with a number of bands 8, 9, 14, 16, 8, 13, 8, and 12 corresponding to SCoT-2, SCoT-4, SCoT-5, SCoT-22, SCoT-26, SCoT-34, SCoT-36 and SCoT-30, respectively.

**Table 7:** The Influence of MN-NPs concentrations on genomic DNA of the common bean by SCoT -PCR. (+ : present, −: absent).

Ser. No	SCoT primers	Total number of bands	Allele size range (bp)	Mono-morphic bands	Poly-morphic bands	(+ ve) Unique bands	(−ve) Unique bands	Polymorphism %	Molecular Size (bp)	T1	T2	T3	T4	T5
1	SCoT-2	8	1610–260	7	1	0	0	12.5%	1610	+	+	−	−	−
2	SCoT-4	9	1500–210	7	2	1	0	22.22%	1500	+	+	+	−	−
									210	+	−	−	−	−
3	SCoT-5	14	2400–250	12	2	0	1	14.28%	870	+	+	−	−	−
									495	+	+	+	+	−
4	SCoT-22	16	3800–240	12	4	0	4	25%	3800	+	+	+	+	−
									3530	+	+	+	+	−
									955	+	+	+	+	−
									870	+	+	+	+	−
5	SCoT-26	8	980–220	6	2	0	2	25%	625	−	+	+	+	+
									220	−	+	+	+	+
6	SCoT-34	13	2165–230	8	5	0	1	38.46%	2165	−	−	+	+	+
									1680	−	−	+	+	+
									1220	−	−	+	+	+
									990	−	+	+	+	+
									645	−	−	+	+	+
7	SCoT-36	8	945–215	7	1	0	0	12.5%	285	+	+	+	−	−
8	SCoT-30	12	2210–280	8	4	2	2	33.33%	2210	−	−	−	−	+
									1975	−	−	−	−	+
									1080	−	+	+	+	+
									885	−	+	+	+	+
Total		88	−	67	21	3	10	−						
Average		11	−	8.375	2.625	−	−	23.86%						

The total number of bands from DNA amplification using SCoT-2, SCoT-26, and SCoT-36 primers were eight bands ranging from 1610 to 260, 980 to 220, and 945 to 215 bp, respectively. Where, SCoT-26 primer had two –ve unique bands at 625 and 220 bp, which disappeared in control (T1) while amplified in all studied concentrations of nanofertilizers.

The amplification of SCoT-4 primer gave nine bands with molecular sizes ranging from 1500 to 210 bp and the polymorphism was 22.22%. In addition, it gave one + ve unique band at molecular size 210 bp for T1. While the amplification of SCoT-5 primer gave a total number of bands (fourteen) which ranged from 2400 to 250 bp with a polymorphism of 14.28%. There is one –ve unique band at 495 bp in the transaction T5 only.

In primer SCoT-22, the total number of bands were sixteen ranging from 3800 to 240 bp with four polymorphic bands at molecular size 3800, 3530, 955, and 870 bp that were present in all transactions except the transaction T5. In addition, the primer SCoT-34 had a total number of bands thirteen ranging from 2165 to 230 bp with five polymorphic bands (38.46% polymorphism). The polymorphic bands had one –ve unique band at 990 bp that disappeared in T1 only.

Finally, the amplification of the SCoT-30 primer gave twelve bands ranging from 2210 to 280 bp with four polymorphic bands (33.33% polymorphism). There had two -ve unique bands at 1080 and 885 bp that disappeared only in T1, also there had two + ve unique at 2210 and 1975 bp in the transaction T5.

### Effect of different MN-NPs concentrations on protein profiles using SDS-PAGE

**Figure 4:**
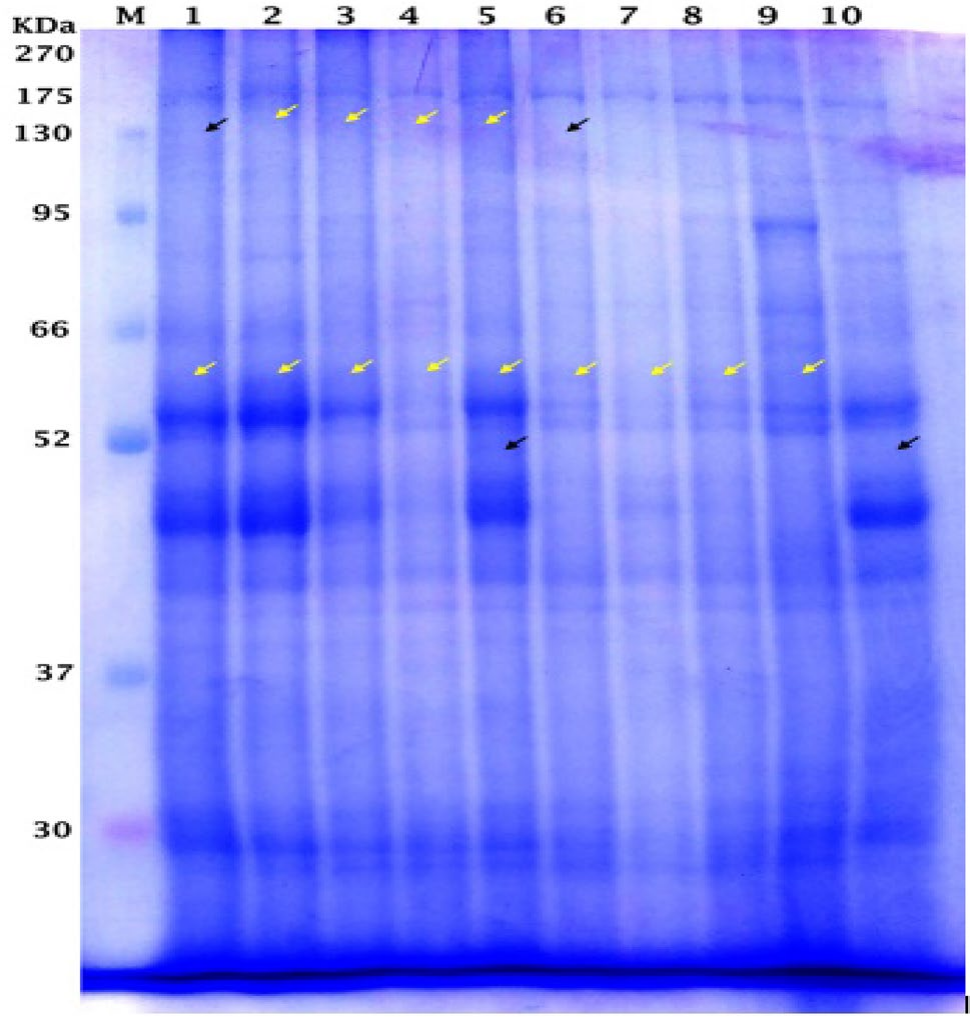
Effect of different concentrations of MN-NPs on protein banding patterns of W.S.P. for common bean in both seedling and flowering stages. Where M is protein marker, 1:5 are T1:5 in seedling stages, while 6:10 are T1:5 in flowering stages

The expression of some genes encoding proteins because of MN-NPs effects on common beans illustrates in Fig. 4 and Table 8. The original figure obtained from protein profiles using SDS-PAGE was illustrated in S4. Twenty-seven bands were obtained in the seedling stage with twenty-three monomorphic bands, while in the flowering stage; twenty-six bands were obtained with twenty-one monomorphic bands. There were four polymorphic bands in the seedling stage but five polymorphic bands were obtained in the flowering stage presented.

**Table 8:**
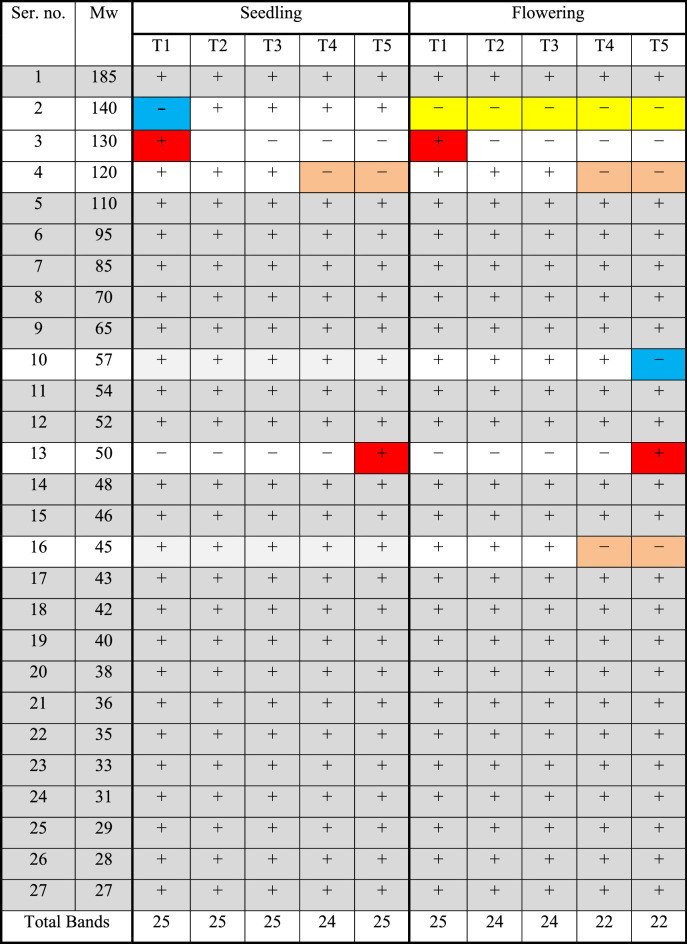
Effect of MN-NPs concentration on electrophoretic of W.S.P. banding patterns of common bean in seedling and flowering stages. (+ : present, −: absent).

The data displayed that both stages were characterized by the presence of protein at Mw 50 Kilo Daltons (KDa) in the transaction T4 only also the protein at Mw 130 KDa present in the transaction T1 only. While the protein at Mw 120 KDa is absent in transactions T3 and T4 only. In the flowering stage, the protein at Mw 57 KDa is absent only in T4, and the protein at Mw 45 KDa is absent in transactions T3 and T4. While in the seedling stage, the protein at Mw 140 KDa is absent in control while present in the other transactions.

### Toxicity test

The common bean seeds obtained from various treatments using the combination of ZnO + MnO_2_ + MoO_3_ -NPs showed that they are not nontoxic. Where the effective concentration (EC50) as obtained from using a Microtox analyzer for all samples was less than 100 indicates they are eco-friendly. This result is in the same direction as the study of Jiang et. al^54^ on the toxicity of silver nitrate (AgNO_3_) and silver nanoparticles (Ag-NPs), who found that Ag-NPs were less toxic than AgNO_3_.

## Discussion

The supply requirement of essential nutrients for the plant at different growth stages is needful to raise the quality and yield of the crop. In this experiment, we applied the essential macronutrients (NPK) as soil fertilizer and different concentrations of micronutrients as a foliar nanofertilizer at vegetative growth stage, which significantly improved common bean growth and yield parameters. The application of micronutrients via foliar spray is important to increase the use efficiency of micronutrients and promote crop yields, Where the absorption of nutrients is faster and supplies the plant with the required amount of nutrients^55,56^. Volkweiss^57^ noted that the foliar application of nanoparticle fertilizing is a buoyant method for using micronutrients since it demands lower rates and micronutrients that are not directly affected by the soil, that being so averting the losses by stabilization. Li et al.^58^ showed that the affirmative impact of the external application of ZnO NPs improved shoot/root biomass of cucumber plant.

The combined MN-NPs affect the physiological processes of the plant. Where they were the reason for improving the vegetative growth characteristics as presented in Tables 3 and 4, which reflected on increasing pods and seeds synthesis per plant, as a result, stimulating plant enzymes and translocation of metabolites from vegetative organs to the seeds. Also, the application of MN-NPs was necessary to improve and stimulate the physiological processes of plants reflected in enhancing the synthesis of photosynthetic pigments content in common bean leaves. The improvement of the content of the photosynthetic pigment enhances the performance of the plants hereby the vegetative growth characteristics were promoted under MN-NPs treatments^59^. Kheirizadeh et al.^60^ reported that the negative effects of salinity on the growth character, yield and nutrients accumulation in barley plants could be reduced by foliar application of nano fertilizer. They found that Zn and Fe in nano-form improve the growth character and yield due to their critical role in biochemical and physiological activities.

Indeed, owing to the size of nanomaterials, they are gradually absorbed and are directly participated in biochemical reactions, improving the plant enzymes' action, reinforcing the exchange of nitrates to ammonia, production of amino acids and enzymes, growing plant transpiration, and photosynthesis operation, plus to improve nitrogen and carbon nutrition, subsequently, nanomaterials have direct effects on mineral nutrition of plants^61–63^**.** Zn stress affects either by metabolic processes or by absorption competition on the plant's content of nutrients. Hossain et al. showed that excess zinc leads to a reduction of iron and magnesium accumulation while the copper content increases. This may be due to that Zn and Cu use the same transporters^64^. Whereas, Feigl et al.^65^ stated that treatment of Brassica by Zn significantly increased the content of Cu and Fe but decreased the content of Mn in the roots in comparison to the control.

Moreover, the MN-NPs played an important role in the movement of metabolites from the leaves to the seeds. Where it is the main reason for the improvement of the content of carbohydrates and lipids in the seeds of the common bean gotten from the treatment T4 as shown in Table 5. Indeed, Zn plays an important role in the metabolism of proteins and carbohydrates^8^, while manganese affects the metabolism of nitrogen^12^, whereas molybdenum is an essential element for the biological fixation of nitrogen (BNF)^16^. The results obtained can be attributed to the interaction of nanomaterials with plants which results in different morphological and physiological in plants, according to the characteristic of the nanomaterials. Distinctive characteristics such as size, form, chemical structure, and active dosage affect on the implementation of nanomaterials. The favorable and unfavorable influences of nanomaterials on plant expansion depend on the size, composition, concentration, and physical and chemical properties of nanomaterials as well as the plant^20,30^. These consequences were consistent with those got from SCoT-PCR analysis (Fig. 3 and Table 7). The difference in proximate components and minerals in leaves and seeds of common bean plant may be due to the genetic mutation on genomic DNA. Where new bands were appeared and other were disappeared in the SCoT-PCR analysis as effect of the various concentration of MN-NPs on the plant.

Start codon targeted (SCoT) marker is a new method based on the short-conserved region in plant genes surrounding the translation inception codon or beginning codon^49^. It is longer than other markers where it consists of 18-mer, which is connected to functional genes and their corresponding characters^66^. The alterations in the priming places lead to changes in the homologous recombination and annealing conditions that give rise to the evanescence of specific bands and the occurrence of novel bands^67^**.**

The data in Table 7 displayed that the more effective concentration of MN-NPs on the genomic DNA was 40 mg/l compared to the control. Some bands vanished and others were evidenced in all studied transactions compared to control, this might be described by the uppermost concentration of nanofertilizer acting as a genotoxic agent producing DNA damage in the priming locations. This agrees with Ghosh et al.^68^, who detected that high concentrations of ZnO-NPs lead to a rise in the chromosome aberrations of Allium cepa and break the DNA strand. Also, as the concentration of ZnO-NPs was growing the content of ROS was increased in Vicia faba and Nicotiana tabacum, this leads to ROS reaching genomic DNA and causing DNA strand damage.

The genotoxicity effect as a result of the change in the frequencies of bases pair mutation could be qualitatively determined by studying the genomic template stability percentage (GTS%), which relates to the degree of DNA variation^69^. Table 9 illustrates that the values of GTS% which are reduced with raising the concentration MN-NPs indicate that all MN-NPs concentrations had a significant effect on the plants' genomic DNA. Previous studies found that raising the implementation of Ag-NPs on the tomato up to 80 mg/l^70^, ZnO-NPs on the fava bean plant up to 50 mg/l^71^, and MoO_3_-NPs on common bean up to mg/l^36^ caused decreasing the GTS%.

**Table 9:** Number of new appeared ( +) and disappeared (−) bands as related to control and GTS % in common bean plants under the effect of MN-NPs using eight SCoT primers.

Ser No	Primer name		Treatments				
			T2	T3	T4	T5	T1
1	SCoT-2	+	−	−	−	−	8
		−	−	1 (1610 bp)	1 (1610 bp)	1 (1610 bp)	
2	SCoT-4	+	−	−	−	−	9
		−	1 (210 bp)	1 (210 bp)	2 (1500, 210 bp)	2 (1500, 210 bp)	
3	SCoT-5	+	−	−	−	−	14
		−	−	1 (870 bp)	1 (870 bp)	2 (870, 495 bp)	
4	SCoT-22	+	−	−	−	−	16
		−	−	−	−	4 (3800, 3530, 955, 870 bp)	
5	SCoT-26	+	2 (625, 220 bp)	2 (625, 220 bp)	2 (625, 220 bp)	2 (625, 220 bp)	8
		−	−	−	−	−	
6	SCoT-34	+	1 (990 bp)	5 (2165, 1680, 1220, 990, 645)	5 (2165, 1680, 1220, 990, 645)	5 (2165, 1680, 1220, 990, 645)	13
		−	−	−	−	−	
7	SCoT-36	+	−	−	−	−	8
		−	−	−	1 (285 bp)	1 (285 bp)	
8	SCoT-30	+	2 (1080, 885 bp)	2 (1080, 885 bp)	2 (1080, 885 bp)	4 (2210, 1975, 1080, 885 bp)	12
		−	−	−	−	−	
Total number of bands			6	12	14	21	88
% of GTS			93.18%	86.36%	84.1%	76.14%	100%

The effect of MN-NPs on the expression of some genes encoding proteins of common bean may be owing to some metabolic processes in plants that had been affected by nanomaterials application^72,73^. This is because of the absorption, accumulation, and translocation of nanomaterials in plants, moreover these particles may be interacting with plant cells causing a modification of gene expression^74^.

Many researchers illustrated that nanoparticles are up to 40 times less toxic than salts and that are more efficient^63,75^. Moreover, Shaban et al. studied the effect of plants fertilized by nano-fertilizer on the rats’ health. They found that the common bean fertilized either by ZnO^76^ or by MoO_3_-NPs^77^ does not cause any harm to rats that feed on it.

## Conclusions

This study illustrated that using the combination of MN-NPs (ZnO, MnO_2_, and MoO_3_) at the concentration 40 mg/L of each one improves the vegetative growth characters of common bean plants as well as enhances the seeds production to 2.24 and 2.34 t/ha during two seasons 2019 and 2020, respectively. We can recommend that the combination of MN-NPs with the concentration of 40 mg/L could be sprayed on common bean plants to ameliorate their growth, yield, genetic, and some quality characters. It can be deduced that micronutrient in the form of nanoparticles have a hopeful future as a nano-fertilizer in the cultivation strip for applications based on nano-biotechnology.

## Supplementary Information

Supplementary Information.

## Data Availability

The datasets used and/or analyzed during the current study are available from the corresponding author upon reasonable request.
