# Supplementary material for: The impact of nanofertilizer on agro-morphological criteria, yield, and genomic stability of common bean (Phaseolus vulgaris L.)
Source: Sci Rep. 2022 Nov 3;12:18552. doi: 10.1038/s41598-022-21834-9 (PMC9633613; doi:10.1038/s41598-022-21834-9)
Supplement: Supplementary file 1 — Supplementary Information. [file 41598_2022_21834_MOESM1_ESM.docx]

**The impact of nanofertilizer on agro-morphological criteria, yield, and genomic stability of common bean (*Phaseolus vulgaris* L.)**

**Dina M. Salama^1,*^, M. E. Abd El-Aziz^2,*^, Essam A. Shaaban^3^, Samira A. Osman**^4^**, Mohamed S. Abd El-Wahed^5^**

^1^ Vegetable Research Department, National Research Centre, 33 El Bohouth St., Dokki, Giza, P.O. 12622, Egypt.

^2^ Polymers & Pigments Department, National Research Centre, 33 El Bohouth St., Dokki, Giza, P.O. 12622, Egypt.

^3^ Pomology Department, National Research Centre, 33 El Bohouth St., Dokki, Giza, P.O. 12622, Egypt.

^4^ Genetics and Cytology Department, National Research Centre, 33 El Bohouth St., Dokki, Giza, P.O. 12622, Egypt.

^5^ Botany Department, National Research Centre, 33 El Bohouth St., Dokki, Giza, P.O. 12622, Egypt.

**The corresponding author**: Dina M. Salama, **Email:** [dinasalama@ymail.com](mailto:dinasalama@ymail.com), Mahmoud E. Abd El-Aziz, Email: [Mahmoud_essam12@yahoo.com](mailto:Mahmoud_essam12@yahoo.com)

**Table S1:** Physical and chemical properties of experimental soil (Combined data of two seasons).

| **Physical properties** | | | | | |
| --- | --- | --- | --- | --- | --- |
| **Texture** | **Clay** | **Silt** | **Sand** | **EC (ds/m)** | **pH** |
|  | **(%)** | | |  |  |
| Clay | 49 | 38 | 13 | 0.49 | 7.41 |
| **chemical properties** | | | | | |
| **Cations (meq/L)** | | | **Anions (meq/L)** | | |
| **Ca** | **Mg** | **Na** | **SO_4_** | **CL** | **HCO_3_** |
| 2.1 | 0.6 | 1.0 | 3.2 | 1.2 | 1.3 |
| **Macronutrient** | | | **Micronutrient (mg/kg)** | | |
| **N (meq/L)** | **P (ppm)** | **K (ppm)** | **Zn** | **Fe** | **Mn** |
| 47 | 25 | 367 | 1.11 | 5.71 | 0.23 |

**Fig. S2.** Average weekly temperature during two seasons 2019 and 2020

|   SCoT-2 |   SCoT-4 |
| --- | --- |
|   SCoT-5 |   SCoT-22 |
|   SCoT-26 |   SCoT-34 |
|   SCoT-36 |   SCoT-30 |
| **Fig. S3**: The effect of MN-NPs concentrations on the genomic DNA for common bean by SCoT-PCR. | |


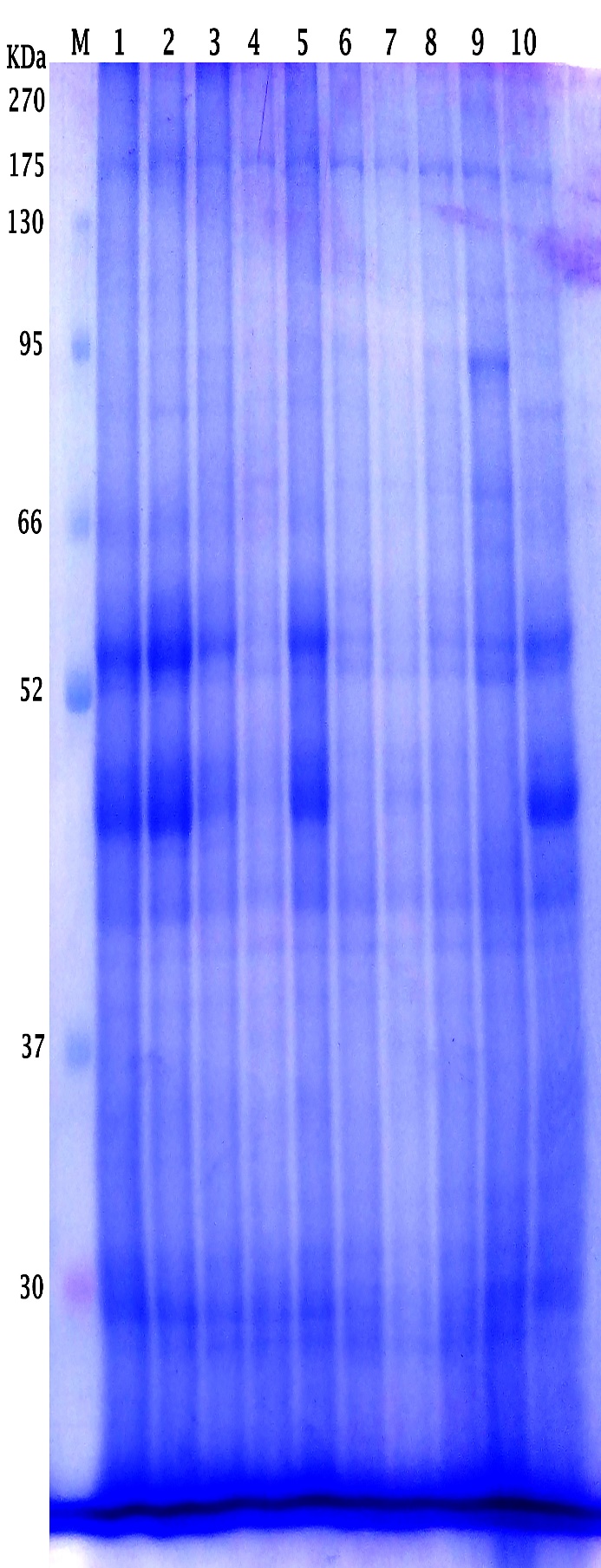

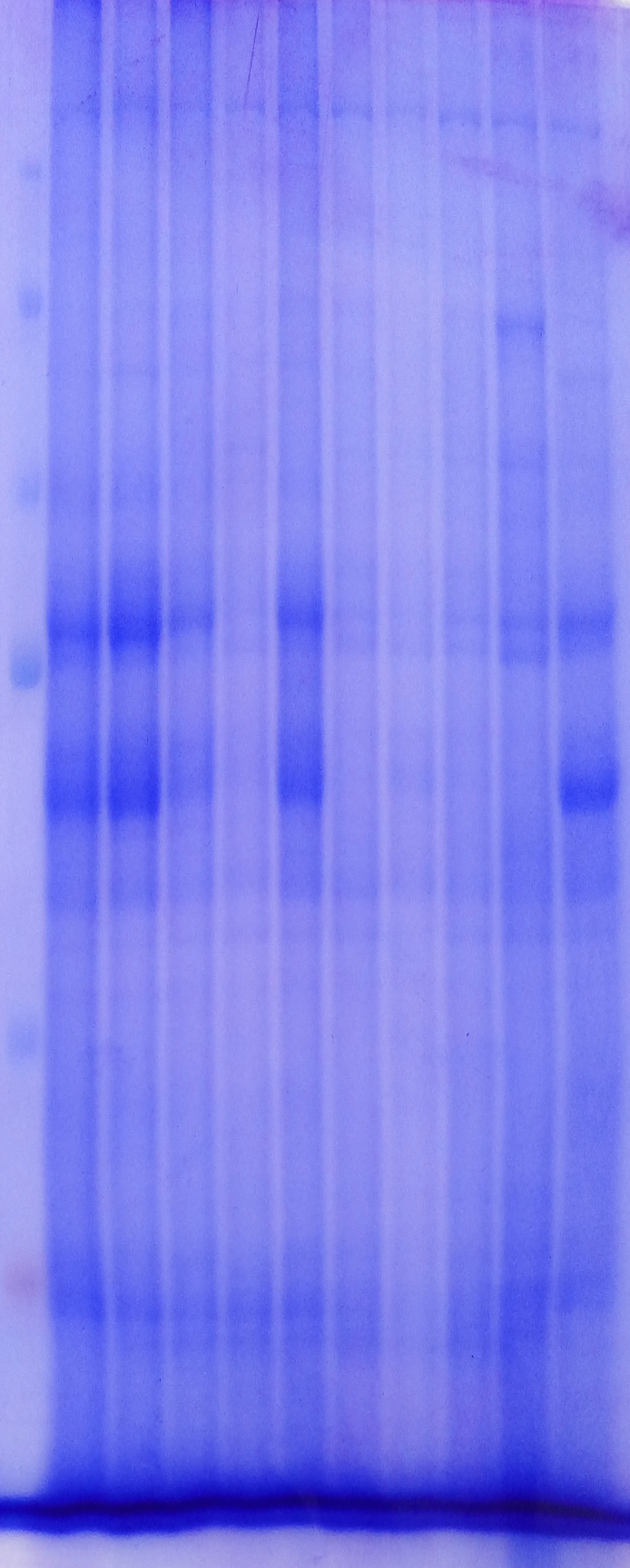


**Fig. S4.** Effect of different concentrations of MN-NPs on protein banding patterns of W.S.P. for common bean in both seedling and flowering stages
